# The Moroccan medical file between practice and politics: a cross-sectional study

**DOI:** 10.11604/pamj.2021.38.153.16330

**Published:** 2021-02-10

**Authors:** Naouadir Rajae, Cheikh Amine, Ajaja Mohamed Rida, Bouatia Mustapha, Naji Saida, El Hassani Amine

**Affiliations:** 1 Mohammed V University, Faculty of Juridical, Economic and Social Sciences, Mohammed Ben Abdallah Avenue Ragragui Al Irfane, Zip code 6430, Rabat Institutes, Rabat, Morocco,; 2 Abulcasis University, Faculty of Pharmacy, Cheikh Zaid Hospital, Department of Pharmacy, Al Irfane City, Hay Ryad, Allal Al Fassi Avenue, Zip code 6533, Rabat, Morocco,; 3 Abulcasis University, Faculty of Medicine, Cheikh Zaid Hospital, Department of Cardiac Surgery, Al Irfane City, Hay Ryad, Allal Al Fassi Avenue, Zip code 6533, Rabat, Morocco,; 4 Mohammed V University, Faculty of Medicine and Pharmacy, Children's Hospital, Ibn Sina University Hospital, Rabat, Morocco; 5 Mohammed V University, Faculty of Medicine and Pharmacy, Cheikh Zaid Hospital, Department of Pediatrics, Al Irfane City, Hay Ryad, Allal Al Fassi Avenue, Zip code 6533, Rabat, Morocco

**Keywords:** Patient’s medical record, computerization, traceability

## Abstract

**Introduction:**

the medical file is a key element of quality reflecting good hospital management. Many steps have been taken through its history leading up to computerization. This Process allows the sharing of files with both the health staff and patients, while respecting the professional confidentiality between parties. However, in Morocco, as is the case in other countries that are unable to computerize all their hospitals, it is necessary to study first the medical file in paper before proceeding with its computerization. The purpose of our study is to describe the state of the hard copy medical record in our Host University and international hospital, Cheikh Zaid in Morocco.

**Methods:**

that is a cross-sectional study that lasted for three months in Cheikh Zaid hospital. The collection of data from this institution was based on the evaluation of 100 medical records of inpatients, seeing as they respond to our study criteria and requirements better than outpatients. Said evaluation was inspired by a clinical audit grid recommended by the High Authority for Health (HAS). Extraction of the results is done using the SPSS 13.0, Microsoft Excel, and Microsoft Visio software. In addition, we used the observation method to correct the errors found.

**Results:**

the results showed that 75% of the files are in good condition and well organized. However, administrative documents were missing in 70% of the cases (national identity card, health insurance card and copy of the patient's consent form). Moreover, in 83% of cases, the identity of the person to be notified in case of complications and the consent form were missing. It is also the case for the discharge report. The latter is incomplete in 97% of the cases. Also, the file transfer data from one service to another were missing in 82% of the medical files.

**Conclusion:**

according to the results, improving the medical file is necessary both administratively and medically. Thus, all parties, including doctors and nurses must be aware of their tasks and roles in this process. Despite the advances in the computerization of the medical file in several hospitals in Morocco, the maintenance of the hard copy version remains unavoidable and still necessary, to protect the rights of both the patient and his medical staff.

## Introduction

In Morocco, preserving the patient's medical record (PMR) is an ethical obligation mentioned in articles 22, 24 and 60 of the medical doctor Code [1] and in Article 44 of the Code of Dentists [2]. It became legal with the promulgation of Law 65-00 on Mandatory Health Insurance (AMO) [3]. A long time ago, the PMR was the simple materialization of a need of the doctor who feared the treason of his memory. Therefore, it is obligatory and essential for each medical institution to establish a PMR, because it´s a multi-faceted tool [4] that represents the integral memory of the physician and combines all of the necessary information and specific data of a patient [5]. Thus, it allows doctors to keep personal notes to not forget anything about the patient's history [6]. On the other hand, the PMR represents a key element in tracing the acts of care carried out by the various health professionals. It is a tool that reflects the quality of care provided to patients, while acting as a tool for planning, reflection, synthesis, and a mean of epidemiological and clinical studies.

In principle, the PMR is established from the first contact between the patient and the health care establishment. The file is updated gradually with each encounter and intervention made by the medical staff in question, and for the duration of the patient´s stay in the hospital [4]. At the end of each contact with a health care institution, the medical file is returned, modified, validated, filed, and then archived [7]. The medical file has a set of elements that entail the following: Administrative file (contains all the elements allowing to identify the patient (identity card or passport), its social security (insurance card), date of entry to the establishment and date of leave [8]); nursing record (contains information specific to nursing practice [9]); professional medical record (as defined by FH Roger-France in 1981: "... a written memory of the clinical, biological, diagnostic and therapeutic information of a patient, both individual and collective, constantly updated." [10]).

In addition, these elements are considered as a trace of each medical act performed. By consensus, these traces are considered as an echo of the quality of care in health institutions. However, Morocco and Tunisia both suffer from a law void concerning the regulation of the content of the medical file compared to France and Belgium, where the file is more detailed because it is very accurate and represents a legislative obligation [11]. For instance, recommendations of health authorities such as the High Authority of Health (HAS) of France, former ANAES (National Agency for Accreditation of Health Assessment) [10] are published on this subject so as to provide health care institutions with direct and clear guidelines, while in Belgium, it is the Royal Decree that determines the minimum general conditions for the content of a medical file in Belgian hospitals [12]. As a result of this juridical gap and based on the principle of computerization of the patient's medical file, the maintenance of a hard copy version is unavoidable in the health establishments. We undertook this work to determine the content of a Moroccan medical file and to evaluate it to find ways to improve it.

## Methods

**Materials:** for this study, we used Microsoft Visio for creating diagrams and the circuit of the medical file that we had traced; SPSS 13.0 for entering and retrieving results; Microsoft Excel for extracting graphics. As for data collection, we used patient records as traceability and control material.

Our study was based on the collection of an estimated 1/3 of the files of outpatients processed per trimester, collected from July to October 2016, with an overall hospital capacity of around 80%. Due to time and resource restrictions, we did not apply the number of subjects´ required (NSR) calculus method because we did not have the prevalence factor. Concerning the discharged patients, the 100 files that have been studied were extracted randomly and without applying any mathematical or statistical formula from the archives, i.e. the surgical wing, Oncology, Cardiology, etc.

On an ethical level, we had the consent of the patients, as well as the permission of the director of the hospital. Furthermore, taking the nature of our study in consideration, and according to the law 28-13, we did not require an ethics board or committee, as this was not an interventional study.

In order to improve the quality of the layout and the contents of a medical file, several methods for evaluation are proposed by the High Authority of Health (HAS) such as: the clinical audit, Quality Improvement Project (QIP or PAQ in French: an approach that allows to analyze the process in order to identify segments that need improvement), Benchmarking, etc. In addition, each institution chooses the most appropriate method according to its available resources as well as its objectives. In our case, we adopted three methods: benchmarking, clinical audit, and observation.

**Benchmarking:** this method consists of comparing the practices of one country with others of the same economic level or more developed. This method aims to draw on foreign experiences to improve the medical record in health facilities in Morocco.

**Evaluation method of medical records:** this is a cross-sectional study of patient records from the archiving service of the Cheikh Zaid hospital, a semi-public multidisciplinary, international university hospital. It offers many services that are categorized as follows: the U department, for the emergencies; the A department for the day hospital, radiology, hospitalization, administration; the B department for external consultation, hospitalization, conference center, laboratory, and workshops center and the C department for medical oncology and radiotherapy.

The collection of data from this institution was based on the evaluation of 100 medical files (as opposed to the minimum determined by the HAS of 30 files), belonging to outpatients by a clinical audit grid inspired by the HAS recommendation, but adjusted to our current environment. In addition, we used the descriptive statistics method to analyze the results. The grid has several instructions to ensure its proper use (Table 1): use one grid per file; numerate each grid chronologically; the patient´s personal information must stay confidential, they should not be mentioned on the grid; each question must be answered by yes or no; the comment section must be used to add further details or notes special to each service and the grid should be adapted to each patient and be specific.

**Table 1:** medical file evaluation criteria

File n°	Medical file evaluation criteria	Yes	No
**General: the conditions of keeping the medical record**	1. The file is in good condition	**◯**	**◯**
	2. The reports are all dated, signed and sealed	**◯**	**◯**
	3. The file is organized, and its elements are well classified (administrative file, clinical file, nursing file, monitoring sheets, summary sheets)	**◯**	**◯**
	4. Is there a file processing and filing service?	**◯**	**◯**
	5. Is there a procedure for accessing the medical record?	**◯**	**◯**
**Administrative file**	6. Patient data is complete		
	◯ First and last name		
	◯ Patient Identification Code		
	◯ File number: H		
	◯ Entry date		
	◯ Data of birth and age		
	◯ Sex		
	◯ Payment method		
	7. Is there a copy of the patient identity card?	**◯**	**◯**
	8. Is there a copy of the patient insurance card (if the patient has insurance)?	**◯**	**◯**
	9. Is there a hospitalization ticket?	**◯**	**◯**
	10. Is there an admission sheet?	**◯**	**◯**
	11. Identity of the person to contact in case of emergency noted in the admission form?	**◯**	**◯**
	12. Identity of the referring doctor?	**◯**	**◯**
	13. Copy of medical care	**◯**	**◯**
	14. Is the consent of medical care is signed by the patient or his next of kin?	**◯**	**◯**
**Clinical record**	15. Is the treating physician mentioned?	**◯**	**◯**
	16. Is the medical admission report filled and completed?	**◯**	**◯**
	17. The summary of consultations is filled?	**◯**	**◯**
	18. Is the full exit report completed?	**◯**	**◯**
	19. Is medical questionnaire filled?	**◯**	**◯**
**Nursing record**	20. Admission data collection sheet in the nursing file is it completed?	**◯**	**◯**
	21. Service/bloc link sheet in nursing file is it completed?	**◯**	**◯**
	22. In case of transfer from a service to another is the transfer sheet filled and completed in the nursing record?	**◯**	**◯**
	23. The patient’s follow-up record in the nursing file is it completed?	**◯**	**◯**
	24. Are the surveillance sheets filled out?	**◯**	**◯**
	25. Exit sheet in the nursing file is it completed?	**◯**	**◯**
**The elements and**	26. Depending on the case, the file must include:		
**data needed to discharge the patient**	◯ The file and the anesthesia sheet		
	◯ The blood transfusion sheets		
	◯ Review report (imaging, biology)		
	◯ Hospitalization report		
	◯ Surgery report		
	27. Release ticket	**◯**	**◯**
	28. Release diagnosis	**◯**	**◯**
	29. Mode and modality of release	**◯**	**◯**
	30. Release date	**◯**	**◯**
	31. checklist	**◯**	**◯**
	32. Drug order after discharge to patient	**◯**	**◯**

**Observation:** after obtaining the results of the clinical audit, we proceeded to observe the practices of health professionals and administrative staff. The purpose of this method was, on one hand, to trace the circuit of the new medical files, and on the other hand, to correct the errors detected on the spot. As it was previously mentioned, we used for this study the SPSS 13.0 software to extract statistical data, and Microsoft Excel to translate them into graphs that will be later analyzed.

## Results

The evaluation of the 100 inpatient medical files belonging to various departments, namely the archives department, as the patients were chosen randomly and from all of the services of the hospital, allowed us to study the composition and to detect the inadequacies of the patient´s medical files. The patient's medical record in our host institution consists of administrative files; clinical files intended solely for the attending physician and/or caregiver; and the nursing care file.

**Figure 1:**
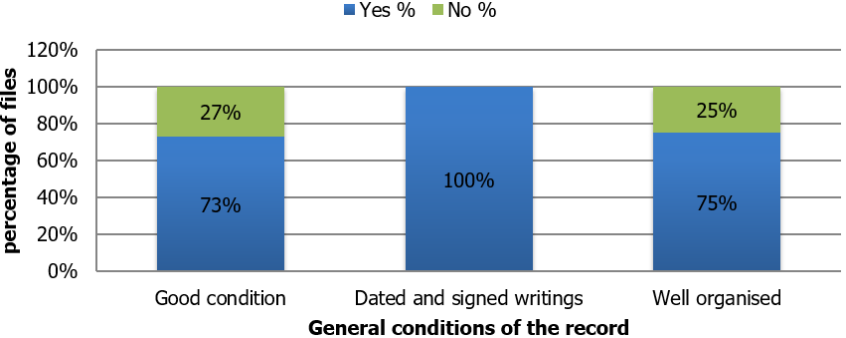
results of the assessment of the patient's medical record

The results have showed that 73% of files are completed under good conditions, 100% are dated and signed and 75% are well organized (Figure 1). However, in parallel with the observation, the results of the evaluation grid allowed us to identify certain problems. Regarding the general presentation of the medical file, we found that the file is most likely to be torn, lost, or in bad condition due to its thickness and the poor nature of the file holder. As a result, some records are torn during their movement from one staff member to another. In addition, some patients are hospitalized for a long time due to the severity of their illness. These patients required several radiology or biology exams while some of them required physiotherapy sessions because of their immobility. In this case, a good follow-up should be arranged to integrate all these elements into the PMR and preserve the medical information of the patient.

**Figure 2:**
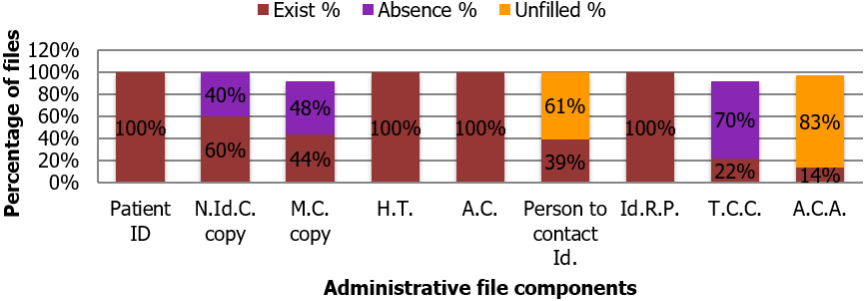
content of an administrative file and the results of the existence and/or absence of its constituent elements

**Administrative file:** the administrative file consists of the following elements: identification of the patient; copy of his National Identity Card (N.Id.C) or passport; copy of an insurance card if the patient has it (M.C); hospitalization ticket (H.T); Admission card (A.C); identity of referring physician (Id.R.P); copy of taking care of charges (T.C.C); Assumption of Care Agreement (A.C.A). It is necessary that the medical file contained initially a copy of the identity card (N.Id.C or CIN in French) as the first identification of a paying patient or one who has medical insurance coverage. In fact, the results of the evaluation have showed that 8% of the files evaluated were for paying patients. In addition, 48% of the files do not contain a copy of the insurance card, to be noted that the latter is essential for the identification of the medical insurance of the patient. (Figure 2).

In certain cases, such as emergency cases, the hospital prioritizes giving proper care and welcoming this person. While waiting for the patient to present proof of their identity, the agents of admission and billing try to extract as many information as possible from the patient´s companion- if there is any. In addition, some patients may end up with multiple files because of missing identity codes when creating their records during their admission to the hospital. This situation may result in a loss of medical information for some patients in the hospital.

**Clinical record:** the clinical file is the essential element in the medical file because it represents the integral memory of the treating physician. In this document, we have found all the doctor's notes as well as his working time. The constituent elements of this file are: a page for the medical admission report that the doctor must write (M.A.R); a summary sheet of the consultations where the doctor must write summaries after each consultation (S.S.C); a report of discharge where the doctor must write his findings when scheduling his patient's discharge (R.D).

**Figure 3:**
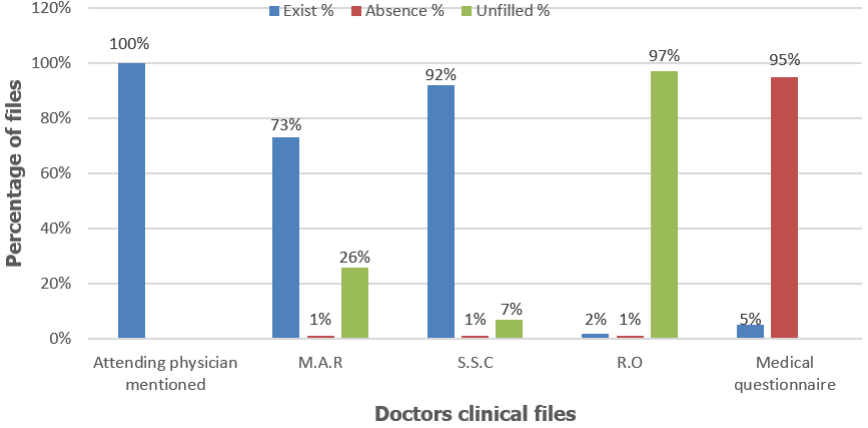
results of the evaluation of the clinical file and the existence of a medical questionnaire

In addition, there is a medical questionnaire separated from the clinical file. The primary purpose of this questionnaire is to establish trust between the patient and his/her doctor, and also to allow the doctor to have all the necessary information about his patient in the event of forgetfulness because of the large number of patients he is treating. During the evaluation of the target samples, only one medical file appeared without a clinical file. In addition, 95% of the medical records did not have a medical questionnaire. Similarly, the part concerned by the report of the patient's discharge was not completed in 97% of the files assessed. However, the results of the other components of the clinical record were generally satisfactory (Figure 3).

**Nursing care file:** nursing care plays an important role in the clinical record. This file consists of: a questionnaire; service/block transfer record; service/service transfer sheet; patient health record sheet; discharge record to be completed when the patient´s leave is scheduled (that is to say the physician scheduled the patient's release after recovery) or unscheduled (that is to say when it is the patient who asked for a leave assuming all responsibility for his state of health). However, each patient has a monitoring record all along his hospital stay. This form is separated from the nursing record, but it too must be completed by nurses only. This sheet is considered as a means of communication between nurses and doctors.

**Figure 4:**
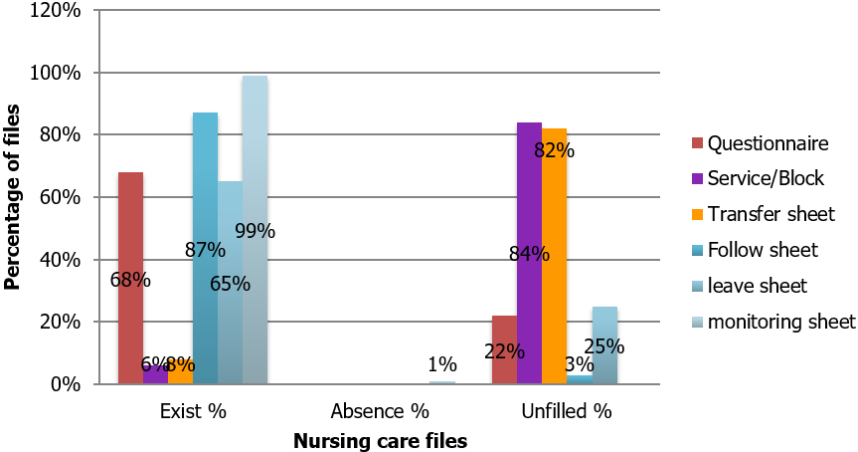
results of nurse care file assessment and the existence of a surveillance record

The results of the evaluation (Figure 4) showed that nurses play a particularly important role in the hospital as well as in the management of medical records. In addition to their care roles, they must ensure the traceability of all medical and paramedical procedures. Moreover, once the file is transferred from the admission service to the hospital service, it becomes the responsibility of the nurses who complete it and keep track of the patient´s care during his hospitalization. In addition, nurses are mediators within a hospital organization; they are the communication link between the patient and his doctor.

## Discussion

Through this study, we found that the file occupies a particularly important place in the process of patient management and hospital management. Our hospital showed that it is trying to follow the French law to ensure good management and a medically accurate medical file in the absence of a similar law in Morocco; this is due to the shared history and academic knowledge, as well as language between the two countries. It is a known fact that most Moroccan doctors choose to deepen their studies abroad, with France as the most popular destination for students to acquire experience, seeing the tight relationship based on collaborations and treaties between the two governments.

Indeed, according to the French experience as well as the study that we conducted, there are no universal or unique medical records per patient [13]. The patient had a specific medical file according to his case and his illness. As a result, the HAS has developed a guide of professional practices in health facilities to evaluate the medical file to improve the quality of its layout and its content [6, 10]. Moreover, based on the experience of developed countries (such as France, Canada, the United States, etc.), the best solution to have a good quality and complete medical file is through computerization [14, 15]. In fact, our country has had an experience of computerization of the medical file which was almost a total success for the first time at the Hassan II University Hospital Center in Fez. On the other hand, our host hospital aims to follow the same approach with its current project for the elaboration of its own computerized medical file. However, and despite the computerization of the latter, the maintenance of a paper file in Morocco is unavoidable, because it is considered as the most solid evidence in Moroccan courts, so we proceeded to evaluate the paper file to improve it.

Through our study, we found that some files play the same role. An example is the report in the clinical file and the record of the hospitalization report, the follow-up card in the nursing care file and the surveillance sheet, etc. In addition, and through observation, we noticed a lack of inter-service communication: the lack of a clear division of tasks (between what is administrative and what is medical); the patient's release before the final diagnosis; no one can judge the doctor if he does not complete his tasks; pressure on the nurses (fulfilling administrative tasks as well as care tasks). We have also noticed that there are no concrete sanctions to be applied if there are any infringements from all sides; the only efforts that have been observed are those of sensitizing the staff and raising awareness among the personnel.

In addition, we found that the patient's medical record does not have a shelf life. However, there is a law that determines the retention period for each medical file in France. Thus, for a good quality of health system, they proceeded to the computerization and sharing of the medical file (health file) with the rights holders. As an added strength, this study allowed, at first, to have an idea about the components of a paper medical file in Morocco, and then identify the problems of its layout and its content and improvement. We found it imperative that a law should be elaborated to determine the constituent elements of a DMP as is the case in France or Belgium, in order to ensure a good maintenance of the DMP paper and to raise awareness for the importance of a good DMP management for genomic, epidemiological and clinical scientific research.

Finally, good hospital management was shown to affect the patient's satisfaction when they return to the hospital to get a copy of their DMP, which our host hospital tries to provide. Furthermore, this study is the first of its kind to be launched in a Moroccan teaching hospital and has showed great promise to the evolution of the tracking and organizing systems in the medical field. Concerning limitations, the biggest obstacle that we have faced is lack of time and the limited resources that we had; seeing as we have occupied the post of intern, we had only three months to finish the study and to present the results, all while not having the ability to speak freely with the medical staff.

## Conclusion

To sum up, the patient's medical file is unlike its administrative counterpart. It is a file that requires higher standards and norms. On one hand, it is a tool of care and traceability, and a tool of pathological studies when it comes to a rare disease; on the other hand, it represents the image and quality of care in a hospital. According to this study, the results revealed discrepancies between the standards adopted by the hospital and the reality of the exercise. Through the study, we could categorize the causes on three levels: lack of awareness, of inter-service communication, and of sanctions. The improvement of quality therefore implies acting and improving these three levels first. As a result, we have found that the good record keeping of the patient requires financial, human, technical and time resources. To effectively work on these elements, the Ministry of Health must develop a general law to follow as the French or Belgian models.

### What is known about this topic

- The patient's medical file is unlike its administrative counterpart;
- Patient's medical file is a tool of care and traceability, and a tool of pathological studies when it comes to a rare disease;
- Patient's medical file represents the image and quality of care in a hospital.

### What this study adds

- There are several discrepancies between the standards adopted by the hospital and the reality of the exercise;
- The causes could be categorized on three levels: lack of awareness, of inter-service communication, and of sanctions;
- The good record keeping of the patient requires financial, human, technical and time resources; it requires also implication of authorities and hospital managers.
